# Supplementary figures and images for: Biocalcification in porcelaneous foraminifera
Source: eLife. 2024 Aug 16;13:RP91568. doi: 10.7554/eLife.91568 (PMC11329275; doi:10.7554/eLife.91568)

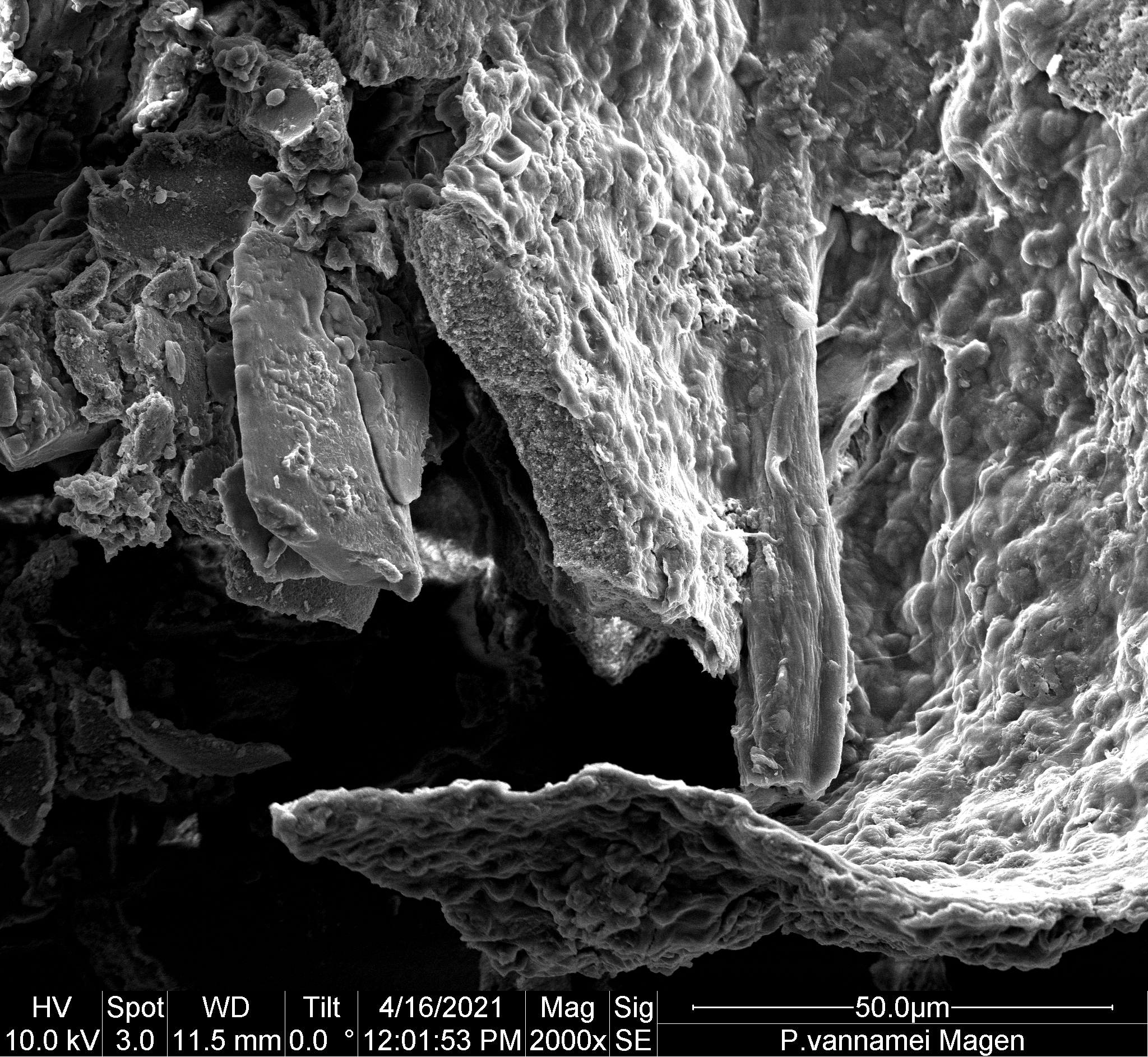

Supplement: Figure 3—figure supplement 1—source data 1. [file elife-91568-fig3-figsupp1-data1.zip › Figure 3-figure supplement 1-source data 6.tif]

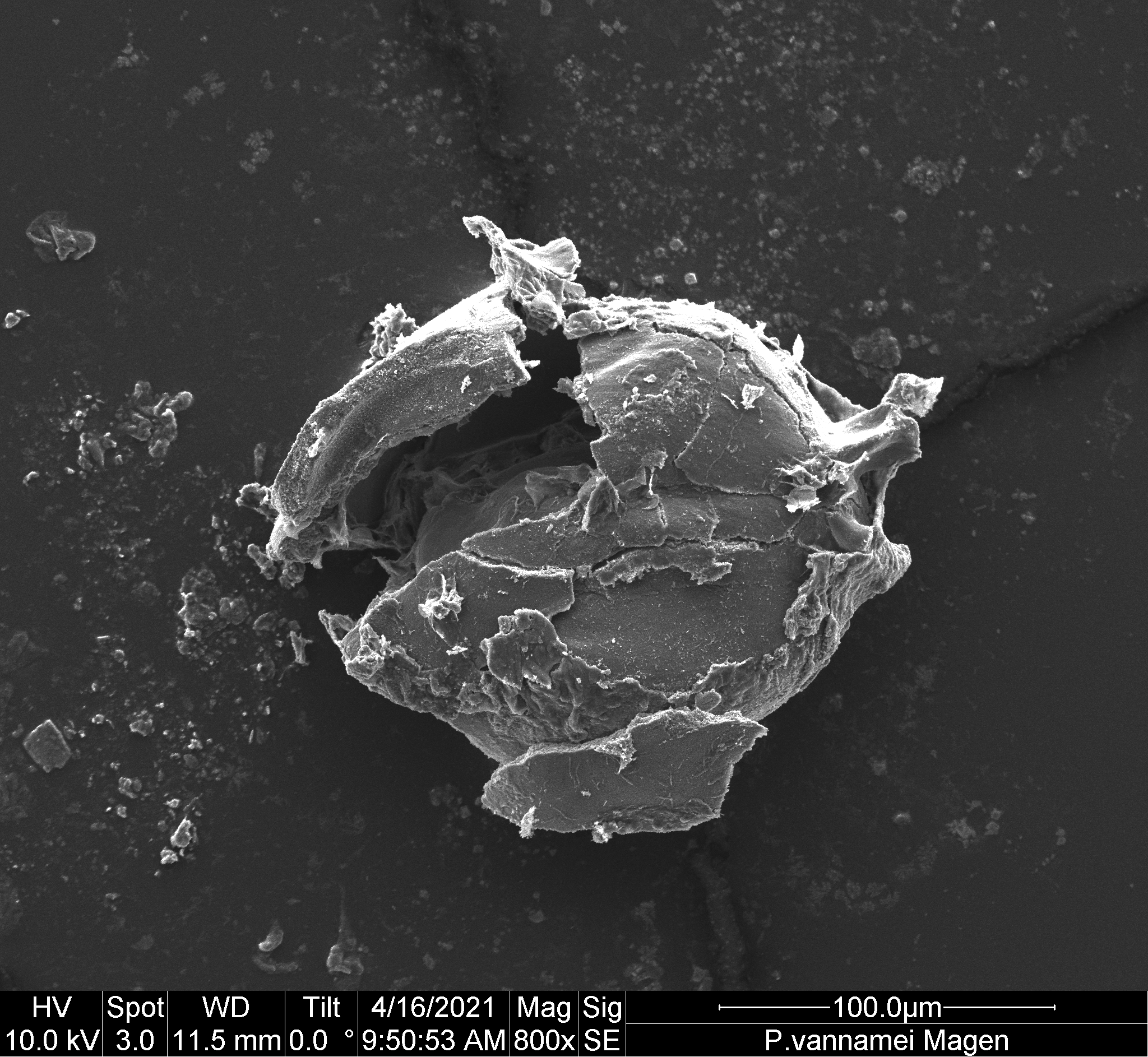

Supplement: Figure 3—figure supplement 3—source data 1. [file elife-91568-fig3-figsupp3-data1.zip › Figure 3 - supplement figure 3 - source data 1.tif]

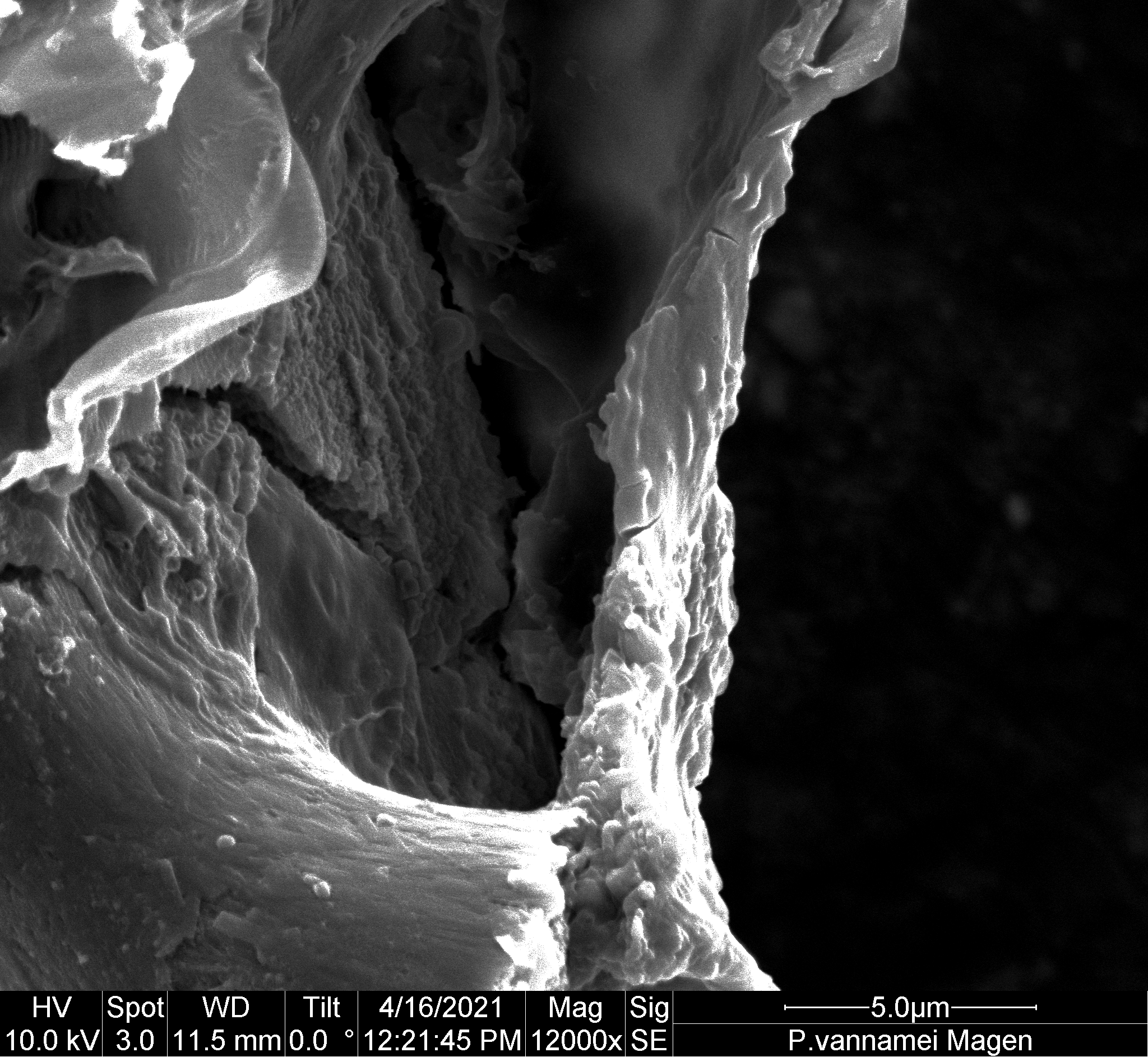

Supplement: Figure 3—figure supplement 3—source data 1. [file elife-91568-fig3-figsupp3-data1.zip › Figure 3 - supplement figure 3 - source data 2.tif]

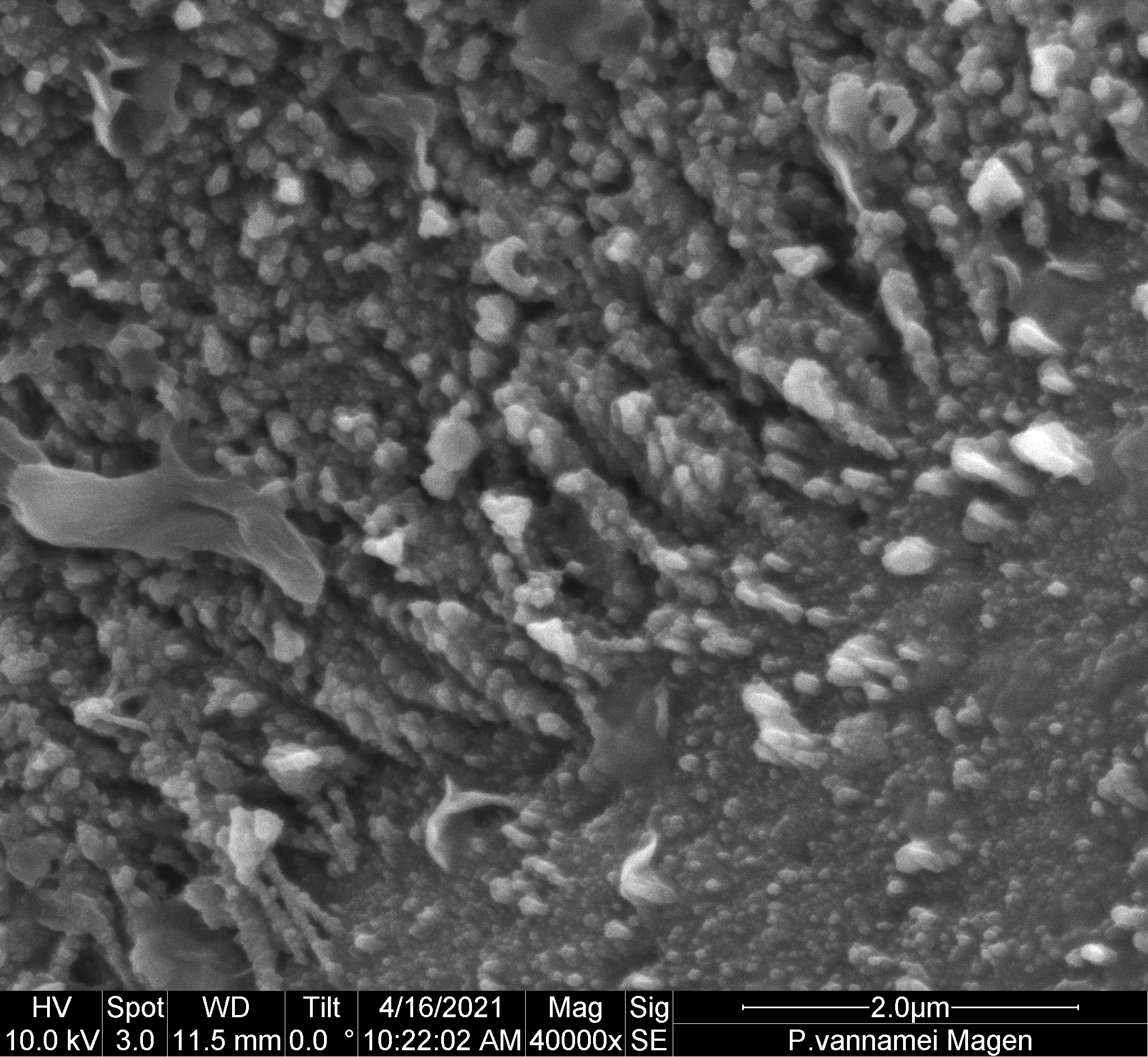

Supplement: Figure 3—figure supplement 3—source data 1. [file elife-91568-fig3-figsupp3-data1.zip › Figure 3 - supplement figure 3 - source data 3.tif]

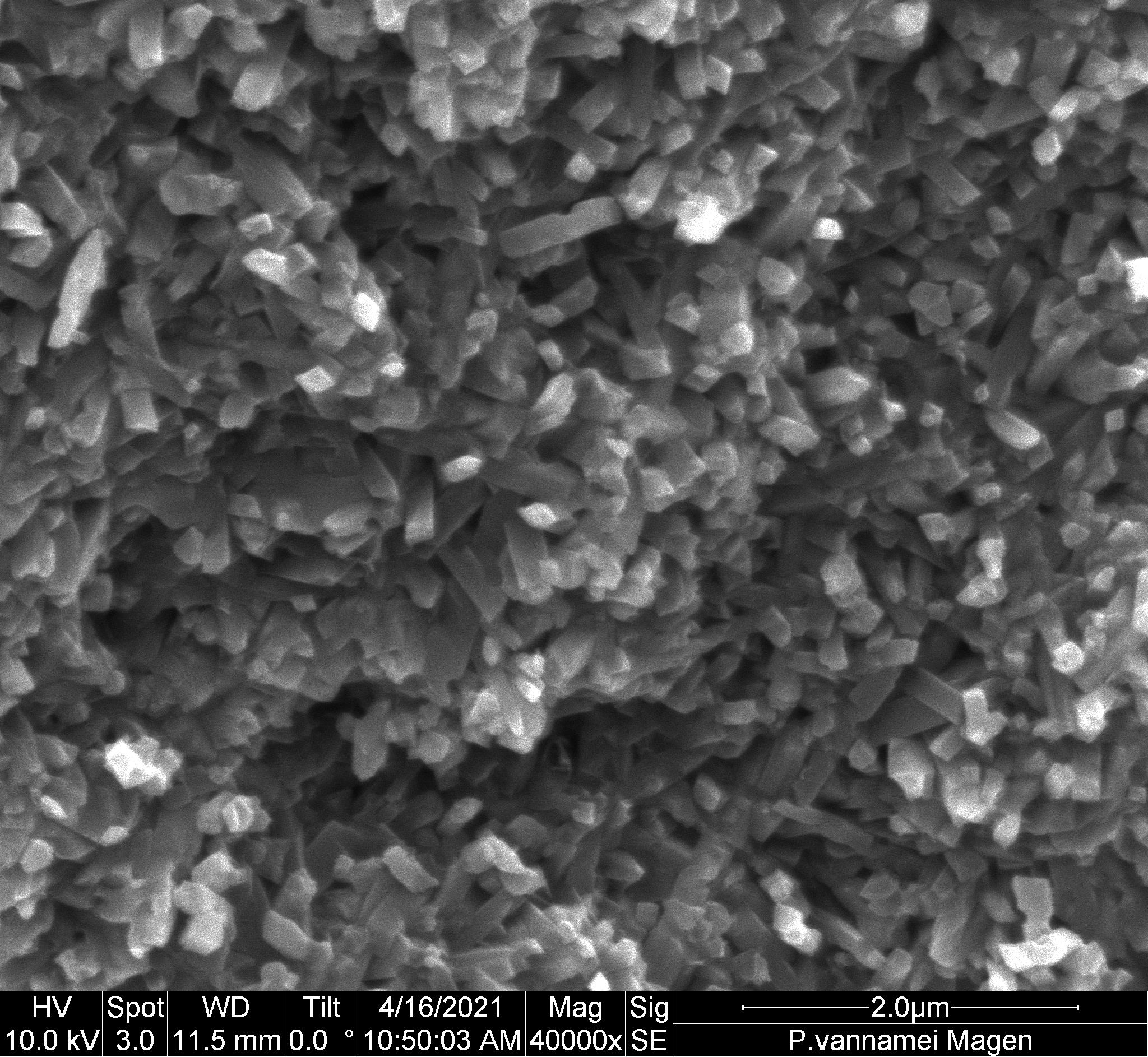

Supplement: Figure 3—figure supplement 3—source data 1. [file elife-91568-fig3-figsupp3-data1.zip › Figure 3 - supplement figure 3 - source data 4.tif]

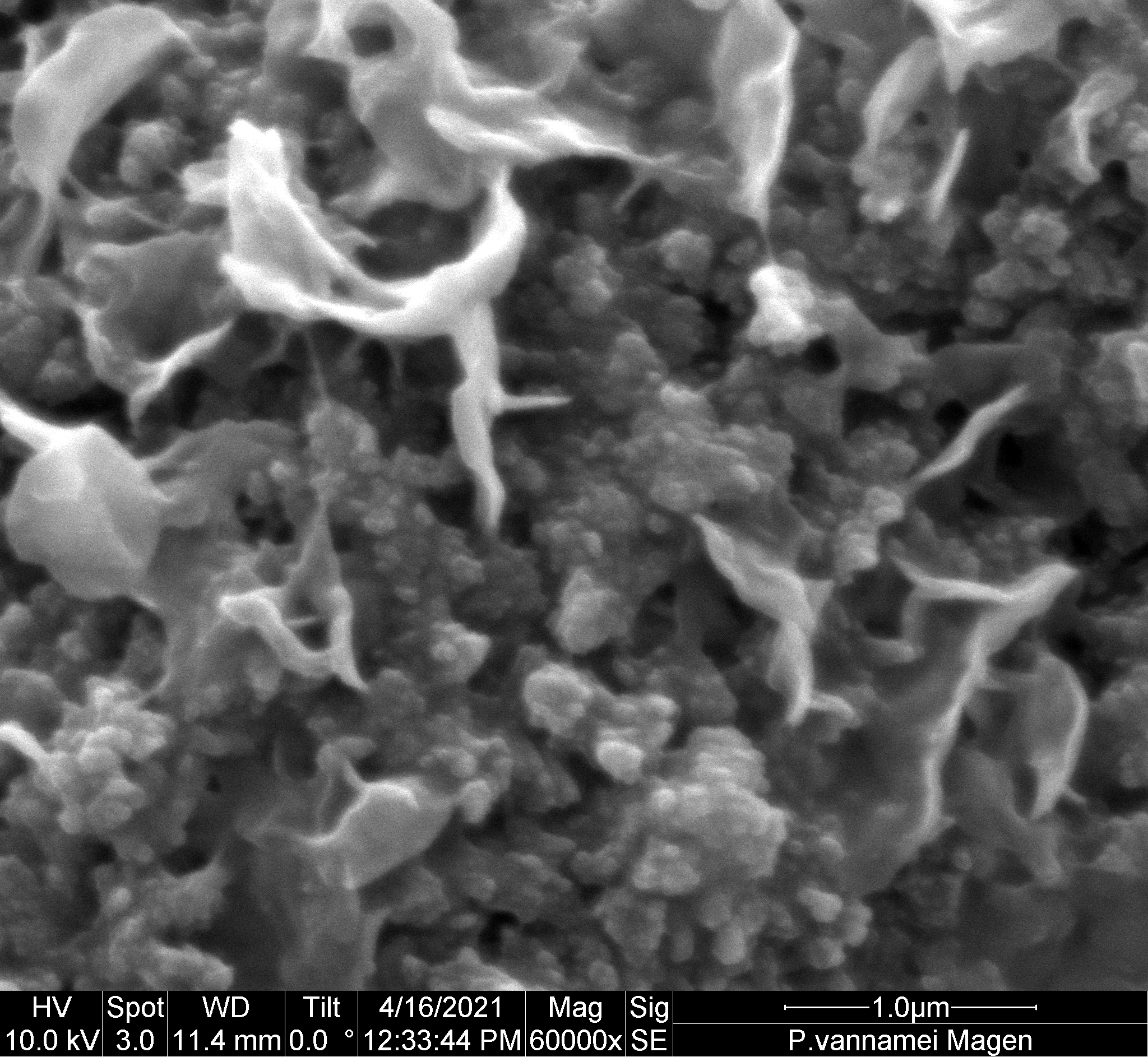

Supplement: Figure 3—figure supplement 3—source data 1. [file elife-91568-fig3-figsupp3-data1.zip › Figure 3 - supplement figure 3 - source data 6.tif]

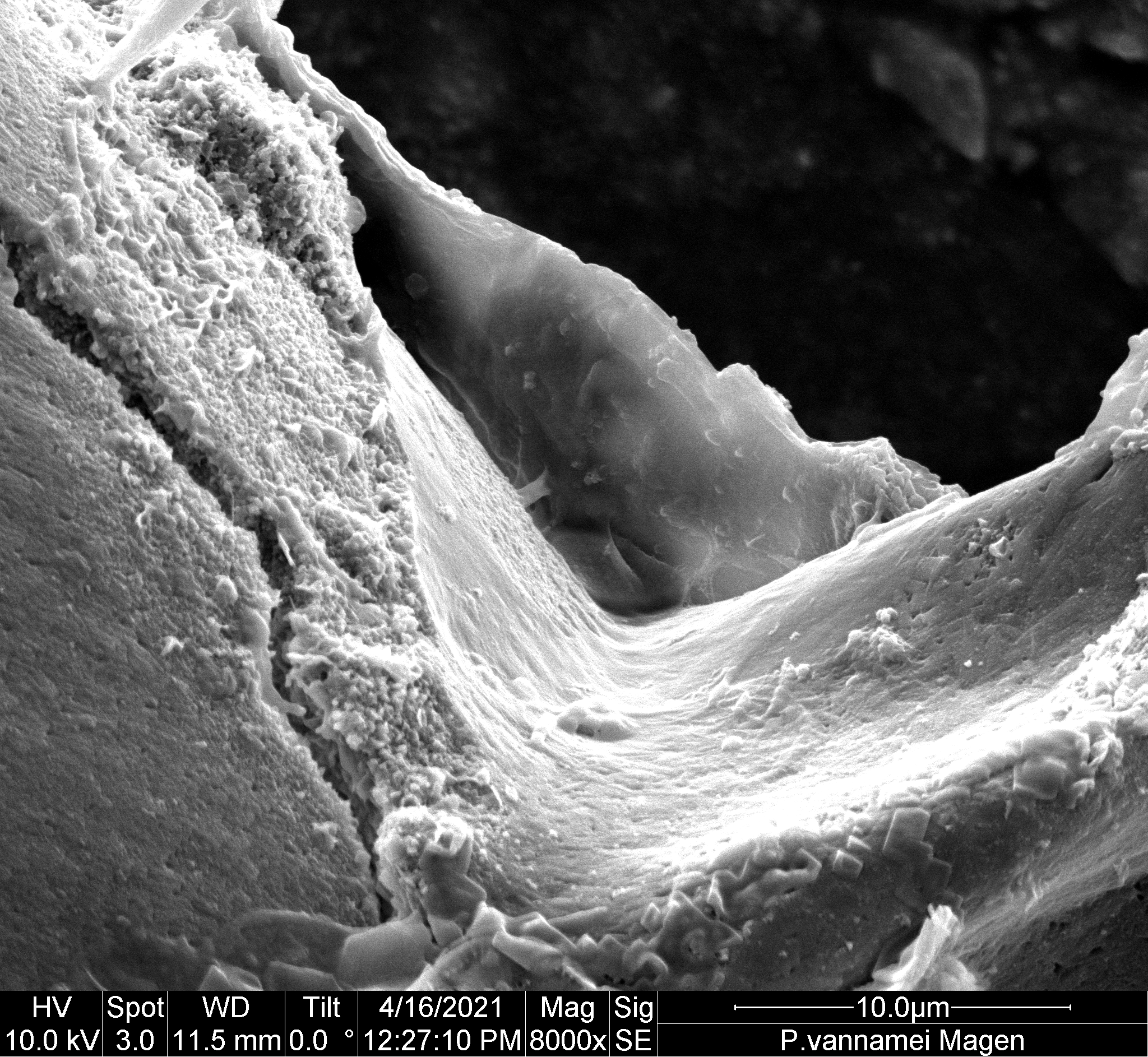

Supplement: Figure 4—source data 1. [file elife-91568-fig4-data1.zip › Source data_Figure 4/Figure 4A-source data.tif]

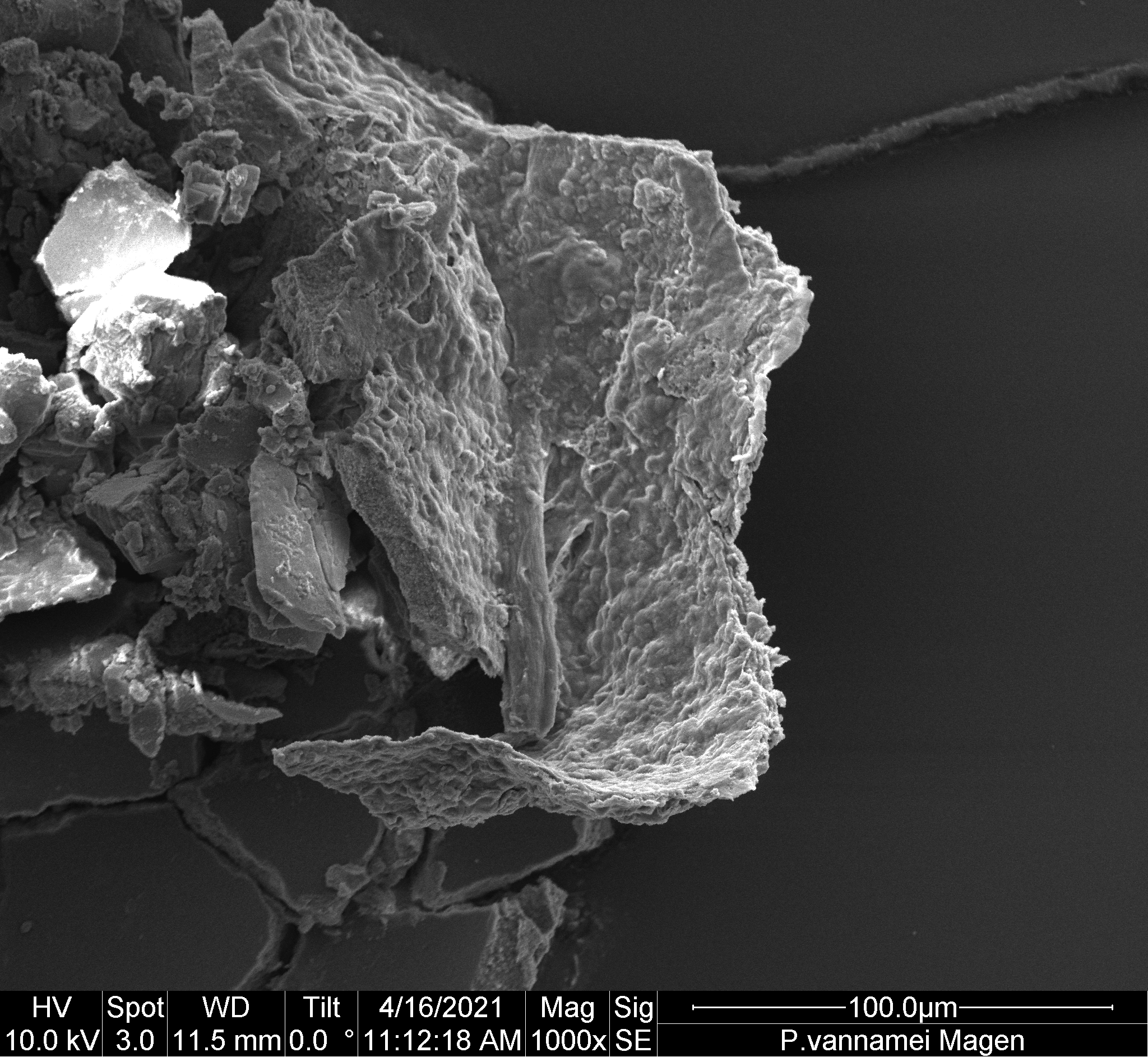

Supplement: Figure 4—source data 1. [file elife-91568-fig4-data1.zip › Source data_Figure 4/Figure 4B-source data.tif]

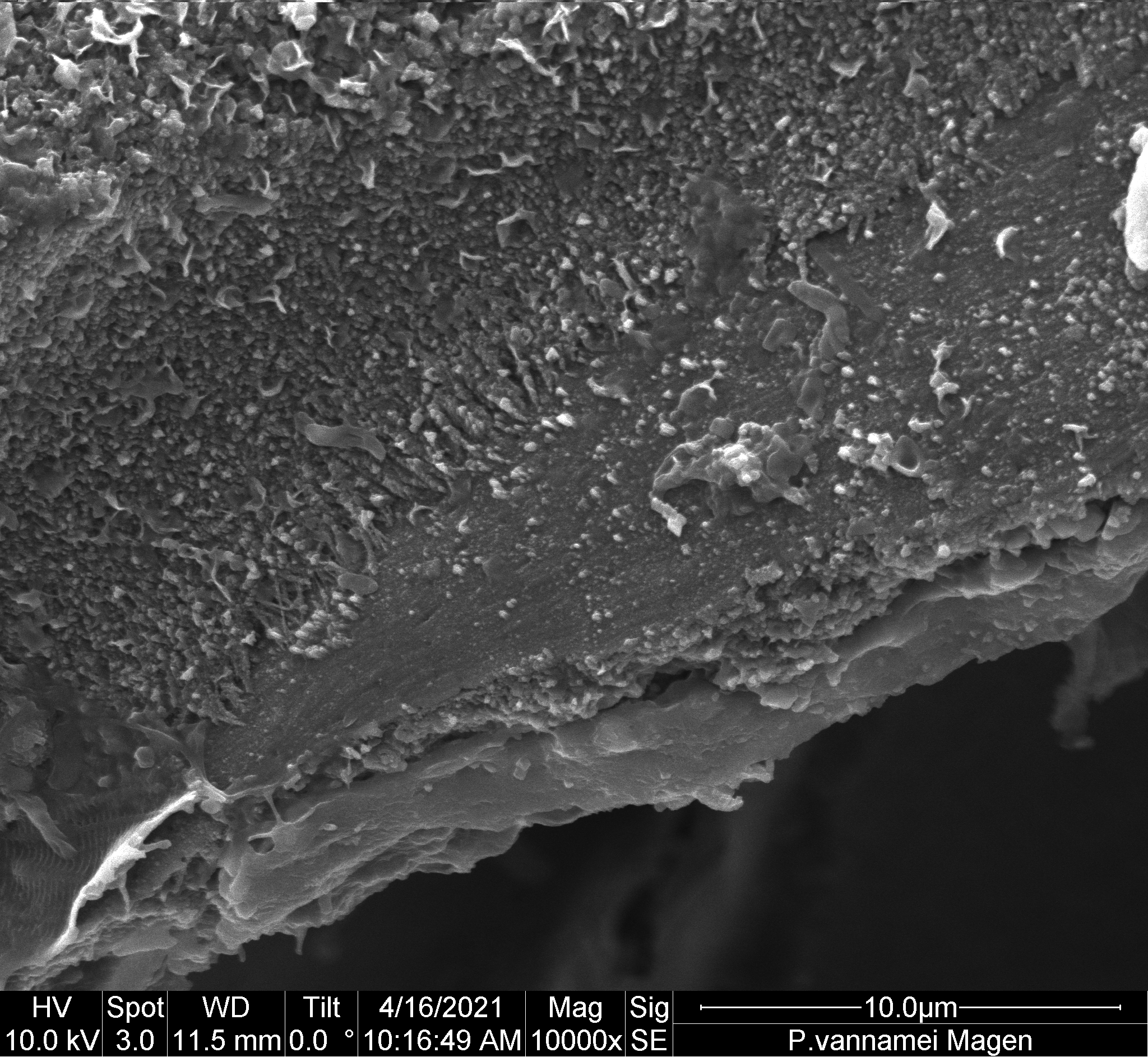

Supplement: Figure 4—source data 1. [file elife-91568-fig4-data1.zip › Source data_Figure 4/Figure 4F-source data.tif]
